# Evaluation of antinociceptive effect of methanolic extract of leaves of *Crataeva nurvala* Buch.-Ham.

**DOI:** 10.1186/1472-6882-14-354

**Published:** 2014-09-24

**Authors:** Md Moniruzzaman, Mohammad Zafar Imam

**Affiliations:** College of Pharmacy, Dongguk University, Goyang, 410-820 Republic of Korea; Department of Pharmacy, Stamford University Bangladesh, 51 Siddeswari Road, Dhaka, 1217 Bangladesh

**Keywords:** Antinociceptive, *Crataeva nurvala*, Capparidaceae, Opioid system, Glutamatergic system, Medicinal plants

## Abstract

**Background:**

*Crataeva nurvala* Buch.-Ham. (Family: Capparidaceae) is widely used as anti-inflammatory, contraceptive, laxative, lithotropic, febrifuge and as tonic in traditional medicine. This study evaluated the antinociceptive effect of the methanolic extract of the leaves of *Crataeva nurvala* (MECN).

**Methods:**

The antinociceptive activity was investigated using heat-induced (hot-plate and tail-immersion test) and chemical-induced (acetic acid, formalin and glutamic acid) nociception models in mice at different doses (50, 100, and 200 mg/kg, p.o.) of MECN. Morphine sulphate (5 mg/kg, i.p.) and diclofenac sodium (10 mg/kg, i. p.) were used as reference analgesic drugs.

**Results:**

MECN produced significant dose-dependent antinociception when assessed using hot plate test, tail immersion test and acetic acid-induced abdominal writhing test (65.55%). Likewise, MECN at similar doses produced significant dose-dependent inhibition in both neurogenic (50.82%) and inflammatory pain (73.53%) induced by intraplantar injection of formalin (2.5% formalin, 20 μl/paw). Besides, MECN also significantly inhibited the glutamate-induced (10 μM/paw) pain in mice (74.68%). It was demonstrated that pretreatment with naloxone (2 mg/kg, i.p.) significantly reversed antinociception produced by MECN in hot plate and tail immersion test suggesting the involvement of opioid receptor. In addition, administration of glibenclamide (10 mg/kg, i.p.), an ATP-sensitive K^+^ channel antagonist could not reverse antinociceptive activity induced by MECN.

**Conclusion:**

The results suggest that MECN possesses antinociceptive activity involving inhibition of opioid system as well as the glutamatergic system supporting its traditional uses.

## Background

*Crataeva nurvala* Buch.-Ham., a common plant in Bangladesh locally known as Borun or Bonna, is a medium sized branched deciduous tree which grows throughout the bank of rivers, canals, lakes and roadsides of tropical, and sub-tropical countries of the world [1]. For a long period of time this plant has been used in the traditional medicine of Bangladesh as anti-inflammatory, contraceptive, laxative, lithotropic, febrifuge and tonic. It is also useful in the treatment of kidney stone, bladder stone, vomiting, gastric irritation and rheumatic fever [2]. Besides, *C. nurvala* is used to treat waste elimination and breathing problems, metabolic disorders, joint lubrication, skin moisture, wound healing, memory loss, heart and lung weakness and weak immune system. The bark is also used in Unani system as appetite promoter and to decrease bile and phlegm secretion [3]. Preliminary phytochemical screening revealed that the leaves contain L-stachydrine, dodecanoic anhydride, methyl pentacosanoate, kaemferol-*O*-α-D-glucoside and quercitin-3-*O*-α-D-glucoside16 [4]. The stem bark, fruit and root contain saponins, flavonoids, sterols and glucosilinates, friedelin, cadabicine diacetate, lupeol, betulinic acid and diosgenin, glucocapparin, triacontane, triacontanol, cetyl and ceryl alcohol, pentadecane, octanamide, 12-tricosanone and friedelin, rutin, quercitin, varunol and β-sitosterol [5]. It was reported that, the compound lupeol isolated from *C. nurvala* stem bark and its ester lupeol linoleate have shown anti-inflammatory activity in complete Freund’s adjuvant induced arthritic rats [6]. Lupeol also increased glutathione and catalase activities that are indicative of its antioxidant properties [7]. Besides, the extract from the stem bark also showed antidiabetic activity in alloxan-induced diabetic rats [8].

To our best knowledge, there is no report about the antinociceptive activity of *C. nurvala* leaves. So the methanolic extract of *C. nurvala* leaves was investigated for it’s potential antinociceptive activity in different experimental models in mice.

## Methods

### Plant material and extraction

The leaves of *C. nurvala* were collected from the road side plants naturally grown at Muradnagar in Comilla district of Bangladesh in October 2012. The collected samples were identified by Bushra Khan, Principal Scientific Officer, Bangladesh National Herbarium, Mirpur, Dhaka, Bangladesh. A voucher specimen (DACB: 37942) has been deposited in the Herbarium for further reference. Powdered dried leaves (250 g) were macerated with 500 ml of methanol with occasional stirring at 25 ± 2°C for 3 days. The extract was then filtered using a Buchner funnel and a sterilized cotton filter. The solvent was completely removed by rotary evaporator and 10.31 g extract (yield 4.12%) was obtained. This crude extract was used for the acute toxicity and antinociceptive activity studies.

### Animals

Swiss albino mice (20–25 g) were collected from Animal Resources Branch of the International Center for Diarrheal Disease Research, Bangladesh (icddr,b). The animals were kept in standard laboratory conditions (relative humidity 55-60%; room temperature 25 ± 2°C; 12 h light/dark cycle) and were provided with standard diet (icddr,b formulated) and clean water *ad libitum* during acclimatization period. The animals were acclimatized to the laboratory environment for a period of 14 days prior performing the experiments. The animals were fasted overnight before the experiments. All the experimental animals were treated following the Ethical Principles and Guidelines for Scientific Experiments on Animals (1995) formulated by The Swiss Academy of Medical Sciences and the Swiss Academy of Sciences. All experimental protocols were approved by the Ethics Committee of Stamford University Bangladesh (SUB/IAEC/12.03).

### Drugs and treatments

The standard drug morphine sulphate (5 mg/kg) used in hot plate and tail immersion tests and diclofenac sodium (10 mg/kg) in writhing and licking tests were administered intraperitoneally 15 min before the experiments while the animals in control group received vehicle (DMSO : H_2_O in 2:1 ratio) orally at the dose of 10 ml/ kg body weight 30 min before the experiments. MECN was dissolved in DMSO and H_2_O in 2:1 ratio respectively and orally administered to the test animals 30 min before the experiments at the doses of 50, 100, and 200 mg/kg body weight in both the chemical- and heat-induced pain models. Naloxone, a non-selective opioid receptor antagonist, was injected intraperitoneally at 2 mg/kg dose 15 min before the administration of morphine sulphate or MECN (50, 100, and 200 mg/kg) to investigate the involvement of opioid receptor system. Besides, an ATP-sensitive K^+^ channel inhibitor, glibenclamide (10 mg/kg) was also injected intraperitoneally to verify the involvement of ATP-sensitive K^+^ channel pathway.

### Acute toxicity test

The test animals were divided into control and three test groups each containing five animals. MECN was administered p.o. at the doses of 1,000 2,000 and 3,000 mg/kg. The mice were allowed food and water *ad libitum* and all animals were observed for toxic symptoms and mortality for the next 72 h [9].

### Antinociceptive analysis

#### Hot plate test

The mice that showed forepaw licking, withdrawal of the paw(s) or jumping response within 15 s on hot plate kept at a temperature of 50 ± 0.5°C were selected for this study 24 h prior to the experiment. Mice were fasted overnight with water given ad *libitum*. The animals were treated with morphine or extract and were placed on Eddy’s hot plate kept at a temperature of 50 ± 0.5°C. A cut off period of 20 s was maintained to avoid paw tissue damage [10]. The response in the form of forepaw licking, withdrawal of the paw(s) or jumping was recorded at 30, 60, 90, and 120 min following the treatment. Then the % MPE was calculated from the latency periods.

#### Tail immersion test

To evaluate the central analgesic property the tail immersion test was performed. This procedure is based on the observation that morphine like drugs prolongs the tail withdrawal time from hot water in mice [11]. One to two cm of tail of the mice pretreated with morphine or MECN were immersed in warm water kept constant at 54 ± 0.5°C. The latency between tail immersion and deflection of tail was recorded. A latency period of 20 s was maintained to avoid tail tissue damage in mice. The latency period of the tail-withdrawal response was taken as the index of antinociception and was determined at 30, 60, 90, and 120 min after the administration of the drug and extract. Then the % MPE was calculated from the latency periods.

#### Acetic acid-induced writhing test

In this test, the mice were treated with drug or extract and then the writhing was induced by injecting 0.6% acetic acid after 15 and 30 min, respectively, at the dose 10 ml/kg body weight. After five minutes the mice were observed and the number of writhing was counted for 30 min [12]. The contractions of the abdomen, elongation of the body, twisting of the trunk and/or pelvis ending with the extension of the limbs were considered as complete writhing.

#### Formalin-induced nociception

Mice were injected with 20 μl of 2.5% formalin solution (equivalent to 0.92% formaldehyde) made up in saline, into the sub-plantar region of the right hind paw 60 min after MECN treatment and 15 min after injection of Diclofenac sodium. Licking of the injected paw was recorded as nociceptive response at 0–5 min (neurogenic phase) and 15–30 min (inflammatory phase) after formalin injection [13, 14].

#### Glutamate-induced nociception

20 μL of glutamate (10 μM/paw) was injected into the ventral surface of the right hind paw of the mice 30 min after MECN treatment and 15 min after injection of Diclofenac sodium. The mice were observed for 15 min following glutamate injection. The number of licking of its injected paw was indicative of nociception [15].

### Analysis of the possible mechanism of action of MECN

#### Involvement of opioid system

The possible participation of the opioid system in the antinociceptive effect of MECN was examined by injecting naloxone hydrochloride (2 mg/kg i.p.), a non-selective opioid receptor antagonist, 15 min prior to the administration of either morphine or MECN. Then the hot plate and tail immersion latencies were sequentially measured at pretreatment, 30, 60, 90 and 120 min with the same cut off time of 20 s for the safety of animals [16].

#### Involvement of ATP-sensitive K^+^ channel pathway

Possible contribution of K^+^ channel in the antinociceptive effect of MECN was evaluated by using the method described previously [17, 18]. The mice were pretreated with glibenclamide (10 mg/kg), an ATP-sensitive K^+^ channel inhibitor, intraperitonially 15 min before administration of either diclofenac sodium or MECN. The mice were challenged with i.p. injection of 0.6% acetic acid, 30 min post-treatment. Following the injection of acetic acid, the animals were immediately placed in a chamber and the number of writhing was recorded for 30 min, starting from 5 min post injection.

### Statistical analysis

The results are presented as Mean ± SEM. The statistical analysis was performed using one way analysis of variance (ANOVA) followed by Dunnett’s post hoc test or Bonferroni’s test as appropriate useing SPSS 11.5 software. A p value <0.05 was considered significant. The results of the tail immersion and hot plate tests were given with percentage of the maximal possible effect (%MPE), which was calculated as follows:


## Results and discussion

### Acute toxicity test

The present study demonstrated that oral administration of the MECN at the doses of 1,000 2,000 and 3,000 mg/kg did not show any mortality, behavioral changes (sedation, excitability) or allergic manifestations during the 72 h observation period after administration. Therefore, it can be assumed that MECN possess low toxicity profile and the LD_50_ is more than 3,000 mg/kg.

### Hot plate and tail immersion tests

The hot plate and tail immersion methods are useful for the evaluation of centrally acting analgesics which are known to elevate the pain threshold of mice towards heat [19]. These tests are useful to determine the involvement of the opioid receptors in the action of the narcotic drugs or other analgesic agents that give effect in this pathway [20]. In our study we have found the maximum effects of the MECN at all doses reached at 30–90 min (Tables 1 and 2). In both heat-induced methods MECN particularly at 100 and 200 mg/kg doses significantly prolonged the latency period ( P < 0.05). The ability of the extract to prolong the reaction latency suggests that the extract is endowed with central analgesic activity.

**Table 1:** **Antinociceptive effect of methanolic extract of** ***C. nurvala*** **leaves, morphine, and reversal effect of naloxone in hot plate test**

Treatment	Dose(mg/kg)	Response time (s) (%MPE)				
		Pretreatment	30 min	60 min	90 min	120 min
Control	0.1 ml/mouse	5.87 ± 0.20	6.30 ± 0.13	6.72 ± 0.09	8.62 ± 0.32	9.34 ± 0.25
Morphine	5	6.67 ± 0.30	12.70 ± 0.51* (45.23)	14.75 ± 1.03* (60.64)	15.70 ± 0.86* (67.77)	15.84 ± 0.94 (68.82)
MECN	50	5.72 ± 0.74	7.84 ± 0.63 (14.90)	8.31 ± 0.43 (18.17)	10.19 ± 0.60 (31.35)	10.37 ± 0.51 (32.58)
MECN	100	6.71 ± 0.48	8.98 ± 0.56** (17.04)	9.66 ± 0.12** (22.16)	13.16 ± 0.89** (48.52)	13.06 ± 1.66 (47.73)
MECN	200	5.83 ± 0.66	9.54 ± 0.21* (26.22)	11.74 ± 0.72* (41.70)	13.86 ± 1.12* (56.69)	13.71 ± 1.78 (55.60)
NLX	2	7.22 ± 0.34	7.05 ± 0.18	6.85 ± 0.13	6.71 ± 0.29	5.30 ± 0.36
NLX + Control	2 + 0.1 ml/mouse	5.93 ± 0.27	6.15 ± 0.10	6.54 ± 0.19	8.01 ± 0.37	8.38 ± 0.69
NLX + Morphine	2 + 5	6.41 ± 0.37	7.25 ± 0.40^a^ (6.24)	7.65 ± 0.16^a^ (9.15)	8.86 ± 0.26^a^ (18.02)	8.96 ± 0.19^a^ (18.82)
NLX + MECN	2 + 50	5.98 ± 0.61	6.31 ± 0.38 (2.37)	7.41 ± 0.22 (10.17)	9.13 ± 0.79 (22.44)	9.71 ± 0.46 (26.59)
NLX + MECN	2 + 100	5.95 ± 0.81	6.49 ± 0.76^b^ (3.80)	7.93 ± 0.46 (14.05)	9.53 ± 0.61 (25.49)	10.08 ± 1.00 (29.36)
NLX + MECN	2 + 200	6.18 ± 0.96	7.04 ± 0.41^c^ (6.25)	8.75 ± 1.02^c^ (18.64)	9.95 ± 1.33^c^ (27.30)	10.14 ± 0.59 (28.69)

Each value is presented as the mean ± SEM (n = 5). MECN = Methanolic extract of. *Crataeva nurvala* leaves; NLX = Naloxone.

*p < 0.001 compared with the control group (Dunnett’s test).

**p < 0.01 compared with the control group (Dunnett’s test).

^a^ *p* < 0.001 compared with the morphine group (Bonferroni’s test).

^b^ *p* < 0.05 compared with the MECN 100 group (Bonferroni’s test).

^c^ *p* < 0.05 compared with the MECN 200 group (Bonferroni’s test).

**Table 2:** **Antinociceptive effect of methanolic extract of** ***C. nurvala*** **leaves, morphine, and reversal effect of naloxone in tail immersion test**

Treatment	Dose(mg/kg)	Response times (s) (%MPE)				
		Pretreatment	30 min	60 min	90 min	120 min
Control	0.1 ml/mouse	1.78 ± 0.17	2.01 ± 0.19	2.44 ± 0.09	2.77 ± 0.30	2.93 ± 0.29
Morphine	5	1.62 ± 0.10	3.11 ± 0.37* (8.13)	3.95 ± 0.27* (12.66)	4.35 ± 0.28* (14.87)	4.17 ± 0.24 (13.88)
MECN	50	1.75 ± 0.11	2.13 ± 0.11 (2.13)	2.32 ± 0.04 (3.16)	2.79 ± 0.19 (5.70)	2.96 ± 0.06 (6.67)
MECN	100	1.91 ± 0.13	2.63 ± 0.33 (3.98)	3.06 ± 0.53 (6.36)	4.15 ± 0.59* (12.37)	4.28 ± 0.59* (13.08)
MECN	200	1.81 ± 0.14	3.03 ± 0.26* (6.67)	3.75 ± 0.17* (10.66)	4.26 ± 0.34* (13.46)	4.58 ± 0.26* (15.20)
NLX	2	1.91 ± 0.11	2.23 ± 0.17	2.29 ± 0.19	2.30 ± 0.17	2.22 ± 0.32
NLX + Control	2 + 0.1 ml/mouse	1.68 ± 0.15	1.97 ± 0.08	2.11 ± 0.10	2.14 ± 0.13	2.27 ± 0.16
NLX + Morphine	2 + 5	1.94 ± 0.18	2.08 ± 0.08 (0.80)	2.52 ± 0.05^a^ (3.26)	2.95 ± 0.14^a^ (5.64)	3.11 ± 0.11 (6.50)
NLX + MECN	2 + 50	1.91 ± 0.16	2.08 ± 0.07 (0.94)	2.11 ± 0.08 (1.12)	2.13 ± 0.32 (1.22)	2.20 ± 0.38 (1.61)
NLX + MECN	2 + 100	1.78 ± 0.11	2.10 ± 0.13 (1.75)	2.21 ± 0.11 (2.38)	2.32 ± 0.04^b^ (2.99)	2.71 ± 0.15 (5.12)
NLX + MECN	2 + 200	1.95 ± 0.11	2.20 ± 1.10 (1.38)	2.41 ± 0.14^c^ (2.56)	2.55 ± 0.07^c^ (3.33)	2.80 ± 0.06^c^ (4.69)

Each value is presented as the mean ± SEM (n = 5). MECN = Methanolic extract of. *Crataeva nurvala* leaves; NLX = Naloxone.

*p < 0.05 compared with the control group (Dunnett’s test).

^a^ *p* < 0.05 compared with the morphine group (Bonferroni’s test).

^b^ *p* < 0.05 compared with the MECN 100 group (Bonferroni’s test).

^c^ *p* < 0.01 compared with the MECN 200 group (Bonferroni’s test).

### Acetic acid writhing test

The acetic acid induced writhing test is widely used for antinociceptive screening and involves local peritoneal receptors (cholinergic and histamine receptor) as well as the mediators of acetylcholine and histamine [21]. In the abdominal tissues acetic acid injection produces peritoneal inflammation, which triggers a response characterized by writhing [22]. Such types of responses are induced by the release of endogenous mediators of pain such as prostaglandins, bradykinine and cytokines (TNF-α, IL-1β and IL-8) that stimulate the nociceptive neurons, which are sensitive to non steroidal anti-inflammatory drugs (NSAIDs) and opioids [23]. In our study, MECN inhibitied the acetic acid induced writhing in a dose dependent manner. MECN at the dose of 200 mg/kg and 100 mg/kg displayed the highest significant (P < 0.001) inhibition of writhing (65.55% and 51.14%) that are comparable to the antinociceptive activity of diclofenac sodium (69.65%) (Table 3). These responses can be described as a typical model of inflammatory pain in which the sensory neurons are depolarized by directly activating a non-selective cationic channel of cutaneous, visceral and other types of peripheral afferent C fibers [24]. Thus, the significant reduction in the number of acetic acid-induced writings by MECN indicates the antinociceptive potential of this plant and confirms its traditional use for the relief of inflammatory pain.

**Table 3:** **Antinociceptive effect of MECN leaf in acetic acid-induced abdominal writhing test in mice**

Treatment	Dose(mg/kg)	Responses	
		Number of writhing (Mean ± SEM)	% Inhibition
Control	0.1 ml/mouse	65.90 ± 2.05	-
Diclofenac sodium	10	20.00 ± 0.79*	69.65
MECN	50	59.10 ± 3.74	10.32
MECN	100	32.20 ± 1.41*	51.14
MECN	200	22.70 ± 0.91*	65.55

Values are expressed as Mean ± SEM (n = 5); * denotes p < 0.001 compared with control group (Dunnett’s test).

### Formalin induced nociception

In formalin test, intraplantar injection of formalin comprises two phases of painful sensitivity. In the first phase the neurogenic pain is caused by direct activation of type C nociceptive nerve endings, releasing neuropeptides such as substance P, among others. On the other hand, the second phase is characterized as inflammatory pain, related to the release of chemical mediators such as histamine, serotonin, bradykinin, prostaglandins and excitatory amino acids, which can be inhibited by painkillers and anti-inflammatory drugs [25–27]. In our study, MECN produced antinociception in both phases of the formalin test, although the effect was more pronounced in the inflammatory phase (Table 4). Therefore, it is reasonable that it has the same antinociceptive activity as central analgesic drugs. Moreover, the antinociceptive effect of MECN on the later phase suggested the antinociceptive potential could involve the anti-inflamatory property.

**Table 4:** **Antinociceptive effect of methanolic extract of** ***C. nurvala*** **leaf in formalin test in mice**

Treatment	Dose (mg/kg)	Licking number		% Inhibition	
		(Mean ± SEM)			
		Early phase	Late phase	Early phase	Late phase
Control	0.1 ml/mouse	143.20 ± 2.11	149.60 ± 3.61	-	-
Diclofenac sodium	10 mg/kg	28.40 ± 1.36*	7.40 ± 0.51*	80.17	95.05
MECN	50 mg/kg	126.60 ± 7.52	87.40 ± 4.50*	11.59	41.58
MECN	100 mg/kg	111.40 ± 4.58*	50.20 ± 2.44*	22.21	66.44
MECN	200 mg/kg	71.20 ± 0.97*	39.60 ± 2.89*	50.28	73.53

Values are expressed as Mean ± SEM (n = 5); * denotes p < 0.001 compared with control group (Dunnett’s test).

### Glutamate induced nociception

In addition, to investigate the involvement of glutamatergic receptors in this antinociception, we used L-glutamic acid to produce nociception. We found that, MECN significantly inhibited the noxious stimuli by L-glutamic acid in a dose dependent manner. These inhibitions are comparable to the reference drug diclofenac sodium, which produces 68.45% inhibition of the pain (Table 5). The 200 mg/kg produced 74.68% inhibition of the nociception that is higher than the effect produced by the reference drug. Thus it can be assumed that the MECN inhibited the excitatory amino acids, PGE_2_ (Prostaglandin E_2_), NO (Nitric oxide), kinins, protons, SP (Substance P) released by the glutamate and more glutamate in the dorsal horn. MECN may be blocked the NMDA (N-methyl- D-aspartate) and non-NMDA receptors as well as inhibiting the release of NO or some NO-related substances that are present in peripheral, spinal and supraspinal sites of action [15, 28].

**Table 5:** **Effect of methanolic extract of** ***C. nurvala*** **leaf in Glutamate-induced nociception in mice**

Treatment	Dose(mg/kg)	Responses	
		Number of licking (Mean ± SEM)	% Inhibition
Control	0.1 ml/mouse	157.20 ± 4.87	0
Diclofenac sodium	10	49.60 ± 1.08*	68.45
MECN	50	59.60 ± 2.25*	62.09
MECN	100	49.00 ± 2.39*	68.83
MECN	200	39.80 ± 3.53*	74.68

Values are expressed as Mean ± SEM (n = 5); * denotes p < 0.001 compared with control group (Dunnett’s test).

### Involvement of opioid system and ATP-sensitive K^+^ channel pathway

To further investigate the participation of opioid receptor system we investigated the effect of naloxone (a non-selective opioid receptor antagonist) against the analgesic activity of MECN in both the hot plate and tail immersion tests. The data obtained showed that the analgesic effect produced by MECN was reversed by naloxone. It was reported that both in tail-immersion and hot-plate tests thermal stimuli influenced the spinal (through μ2 and δ opioid receptors) and the supraspinal (through μ1/μ2-opioid receptors) reflex respectively [29, 30]. Since naloxone succeeds to antagonize the analgesic activity, thus the analgesic activity of MECN seems to be related to opioid receptor. On the other hand, glibenclamide, an ATP sensitive K^+^ channel blocker did not significantly alter the antinociceptive effect of the extract (Table 6), it is speculated that the antinociceptive mechanism may not involve with the opening of ATP sensitive K^+^ channel pathway.

**Table 6:** **Effect of methanolic extract of** ***C. nurvala*** **leaf on Involvement of ATP-sensitive K** ^**+**^ **channel pathway**

Treatment	Dose (mg/kg)	Responses	
		Number of writhing (Mean ± SEM)	% Inhibition
Control	0.1 ml/mouse	73.90 ± 1.61	-
Diclofenac sodium	10	19.90 ± 0.53*	73.07
MECN	50	54.70 ± 1.83*	25.98
MECN	100	29.90 ± 0.66*	59.54
MECN	200	18.30 ± 1.25*	75.24
Glib	10	75.1 ± 2.01	−1.62
Glib + Diclofenac-sodium	10 + 10	36.20 ± 1.77^a^	51.05
Glib + MECN	10 + 50	50.00 ± 1.53	32.34
Glib + MECN	10 + 100	28.00 ± 2.13	62.11
Glib + MECN	10 + 200	20.00 ± 1.34	72.94

Each value is presented as the Mean ± SEM (n = 5). MECN = Methanolic extract of. *Crataeva nurvala* leaves; Glib = Glibenclemide.

*p < 0.001 compared with the control group (Dunnett’s test).

^a^ *p* < 0.001 compared with the Diclofenac sodium treated group (Bonferroni’s test).

## Conclusion

The results of the present study indicates that MECN exhibited significant antinociceptvie activity at all the tested doses in mice. The effect is dose dependent and statistically significant particulary at 100 and 200 mg/kg doses. The reversal effect by naloxone indicates the involvement of opioid and glutamate pathway in the antinociceptive effect of MECN. This indicates the presence of analgesic phytochemical(s) in the plant extract. Further studies are required to carry out antinociceptive activity test using isolated pure compound.
